# Metformin suppresses proliferation and invasion of drug‐resistant breast cancer cells by activation of the Hippo pathway

**DOI:** 10.1111/jcmm.15241

**Published:** 2020-04-12

**Authors:** Jie Liu, Juan Li, He Chen, Ruiqi Wang, Pingping Li, Yi Miao, Peijun Liu

**Affiliations:** ^1^ Center for Translational Medicine The First Affiliated Hospital of Xi'an Jiaotong University Xi'an China; ^2^ Key Laboratory for Tumor Precision Medicine of Shaanxi Province The First Affiliated Hospital of Xi'an Jiaotong University Xi'an China

**Keywords:** drug‐resistant breast cancer, Hippo pathway, Metformin, scribble

## Abstract

Drug resistance limits the clinical efficacy of breast cancer therapies, and overexpression or activation of Yes‐associated protein (YAP) is common in drug‐resistant cancer cells. Thus, inhibition of YAP may reduce resistance to anti‐cancer drugs. Metformin (MET), a first‐line diabetes medication that also has anti‐tumour activities, induces AMP‐activated protein kinase (AMPK), directly phosphorylates YAP and inhibits YAP transcriptional activity. In this study, we determined the effect of MET on the proliferation and invasion of drug‐resistant breast cancer cells and then investigated the underlying molecular mechanism. Our in vivo and in vitro experiments indicated that MET suppressed breast cancer by an AMPK‐independent pathway to decrease YAP nuclear localization. In drug‐sensitive cells, MET activated the Hippo pathway by increasing KIBRA and FRMD6 expression, but this did not occur in drug‐resistant cells. Scribble (SCRIB), a cell polarity protein, was notably down‐regulated in tamoxifen‐ and paclitaxel‐resistant breast cancer cells relative to sensitive cells. We also found that MET suppressed the proliferation and invasion of drug‐resistant breast cancer cells by increasing the expression and cell membrane localization of SCRIB, which enhanced the interaction of SCRIB with MST1 and LATS1, and inhibited YAP nuclear localization and transcriptional activity.

## 1 INTRODUCTION

Breast cancer is the most common malignancy among women globally.^1^ The most appropriate treatment depends on the breast cancer subtype and may include endocrine therapy (eg tamoxifen [TAM] and aromatase inhibitors), chemotherapy (eg paclitaxel [TAX] and docetaxel) and anti‐HER2 agents (eg trastuzumab).^2^ However, drug resistance is a major reason for treatment failure and can lead to cancer recurrence and death. Thus, it is necessary to identify effective treatments that can overcome drug resistance.

Studies of the mechanism of drug resistance in breast cancer have mainly focused on alterations in the expression and signalling of the oestrogen receptor (ER), activation of growth factor receptor (GFR) pathways, cross‐stalk between ER and GFR networks, activation of the PI3K/AKT/mTOR pathway (including PTEN inactivation), activation of NF‐κB signalling and expansion of breast cancer stem cells (BCSCs).^3^, ^4^, ^5^, ^6^ Recent studies have determined that YAP/TAZ overexpression and/or activation was a major reason for drug resistance in breast cancer.^7^, ^8^

Increased consumption and metabolism of glucose is a hallmark of cancer cells that differentiates them from non‐neoplastic cells.^9^, ^10^ Previous studies showed that TAX‐resistant cancer cells had higher rates of glycolysis than TAX‐sensitive cells and thus had increased glucose uptake and lactate production.^11^ Thus, much research has focused on the unique bioenergetic properties of cancer cells when attempting to enhance the efficacy of cancer therapies.

Metformin (MET) is well‐known to stimulate AMP‐activated protein kinase (AMPK) and has been widely used in Europe since 1957 and in the USA since 1994 for treatment of hyperglycaemia. However, MET has AMPK‐dependent and AMPK‐independent effects.^12^, ^13^ Recent studies have examined the potential use of MET in cancer patients to decrease tumour growth, reduce the risk of cancer and improve prognosis.^14^, ^15^ The effect of MET on reducing the drug resistance of breast cancers is not clear. We examined the use of MET on the proliferation and invasion of breast cancer cells that were resistant to TAM or TAX by focusing on changes in the Scribble (SCRIB)‐induced activation of the Hippo pathway.

## 2 MATERIALS AND METHODS

### 2.1 Cell lines and culture

The human breast cancer cell line MCF7 was obtained from Chinese Academy of Science and was cultured according to their recommendations. The TAM‐resistant cell line (LCC2) and the TAX‐resistant breast cancer cell line (MCF‐7/TAX) were derived from MCF7 cells and were cultured as previously described.^16^, ^17^

### 2.2 MTT assay

Cells were seeded into 96‐well plates and then treated with different concentrations of MET, TAX, TAM or their combinations. At indicated times, 0.1 mL of fresh medium containing MTT (0.5 mg/mL) was added, and cells were then incubated at 37ºC for 4 hours. Then, the medium was replaced by 0.1 mL of DMSO and incubated at room temperature for 10 minutes. The absorbance was measured at 490 nm using a microplate reader (PerkinElmer).

### 2.3 Western blotting, immunoprecipitation and antibodies

Cells were added to RIPA lysis buffer containing a mixture of protease inhibitors, and the total protein concentration was estimated using the Bio‐Rad protein assay reagent (Bio‐Rad). Proteins were separated using 8 to 12% SDS‐PAGE and then transferred onto polyvinylidene difluoride membranes (Millipore). The membranes were blocked with 5% fat‐free milk in TBST, incubated with primary antibodies and then incubated with a horseradish peroxidase‐conjugated secondary antibody (Proteintech). Signals were detected using electrochemiluminescence (Bio‐Rad). The level of each protein is expressed relative to that of GAPDH. Monoclonal antibodies against p‐AMPK, APMK, PCNA, cleaved caspase‐3, caspase‐3, KIBRA, FRMD6, Hippo pathway proteins and SCRIB were obtained from Cell Signaling Technology. Anti‐BCL2 was purchased from Santa Cruz Biotechnology, anti‐DLG5 was purchased from Abcam, and anti‐BAX, anti‐Cyclin D1 and anti‐NF2 were obtained from Proteintech. Immunoprecipitation was performed using Dynabeads Protein G (Thermo Fisher Scientific) according to the manufacturer's instructions.

### 2.4 Colony formation assay

Cells were treated with 4 or 8 mmol/L MET for 24 hours and then reseeded in new dishes for 14 days. The cells were fixed using 4% paraformaldehyde (PFA) and then stained with 0.5% crystal violet.

### 2.5 Flow cytometry analysis

Cells were stained with 2.5 µmol/L carboxyfluorescein diacetate succinimidyl ester (CFSE) at 37°C for 30 minutes and then treated with MET. For the apoptosis assay, cells were collected and stained with Annexin V and 7‐AAD using an apoptosis detection kit (BD Biosciences). Data from CFSE staining and the apoptosis assay were analysed by flow cytometry (FCM, BD Biosciences).

### 2.6 Transwell assay

Cell invasion experiments were performed using the Bio‐Coat cell migration chamber (BD Biosciences), which contains a filter with 8‐μm‐diameter pores. Cells re‐suspended in DMEM/BSA medium (3 × 10^5^ cells/500 μL) were added to the upper chamber. DMEM containing 20% FBS was placed in the lower chamber. After 48 hours, the invading cells on the filter were fixed with 4% paraformaldehyde and stained with crystal violet.

### 2.7 Immunofluorescence

The different groups of cells were fixed with 4% PFA for 10 minutes and then permeabilized in 0.5% Triton X‐100 in PBS for 10 minutes at room temperature. They were then blocked with 5% bovine serum albumin (BSA) and 10% horse sera in PBS for 1 hour, incubated with the primary antibody at 4°C overnight and then detected using Alexa Fluor 488‐conjugated secondary antibodies (Invitrogen) and co‐stained with DAPI. The fluorescence signal was measured using a confocal laser scanning microscope (Leica SP5II).

### 2.8 Luciferase assay

Luciferase and Renilla reporters were cotransfected into cells and were analysed using the Dual‐Luciferase^®^ Reporter Assay System and the Dual‐Luciferase^®^ Reporter 1000 Assay System (Promega).

### 2.9 Immunohistochemistry (IHC)

Paraffin‐embedded tissue samples were deparaffinized, rehydrated and subjected to antigen retrieval. The samples were treated with 3% hydrogen peroxide and then with 10% goat serum in TBST at 37°C for 30 minutes. After washing, samples were incubated with the primary antibody at 4°C overnight and then with biotinylated goat anti‐rabbit IgG and horseradish peroxidase‐conjugated streptavidin. The staining was visualized using diaminobenzidine, with haematoxylin as a counterstain. All images were captured using a microscope slide scanner (Leica MP, SCN400).

### 2.10 In vivo tumorigenicity assay

Female BALB/c mice (5 to 6 weeks‐old) were purchased from the Laboratory of Animal Breeding and Research Center, Xi'an, China. A total of 1 × 10^n^ 4T1 cells (murine mammary carcinoma cells) were injected into the fat pad of the fourth breast of each mouse. Then MET (200 mg/kg) and TAM (5 mg/kg) were given by gavage, or TAX (20 mg/kg) was injected intraperitoneally. After 21 days, the mice were killed and tumour and lung tissues were extracted. All experimental procedures were in accordance with a protocol approved by Institutional Animal Care and Use Committee of Xi'an Jiaotong University.

### 2.11 Statistical analysis

Each experiment was performed at least three times independently, and each result is expressed as the mean ± standard error of the mean (SEM) of these independent replicates. The differences between groups were analysed using Student's *t* test with GraphPad Prism version 7.00. A *P*‐value below .05 was considered statistically significant, and significant *P*‐values are indicated as *(*P* < .05), **(*P* < .01) or ***(*P* < .001).

## 3 RESULTS

### 3.1 MET decreases cell proliferation and survival and increases apoptosis of breast cancer cells that are resistant to TAM and TAX

**FIGURE 1:**
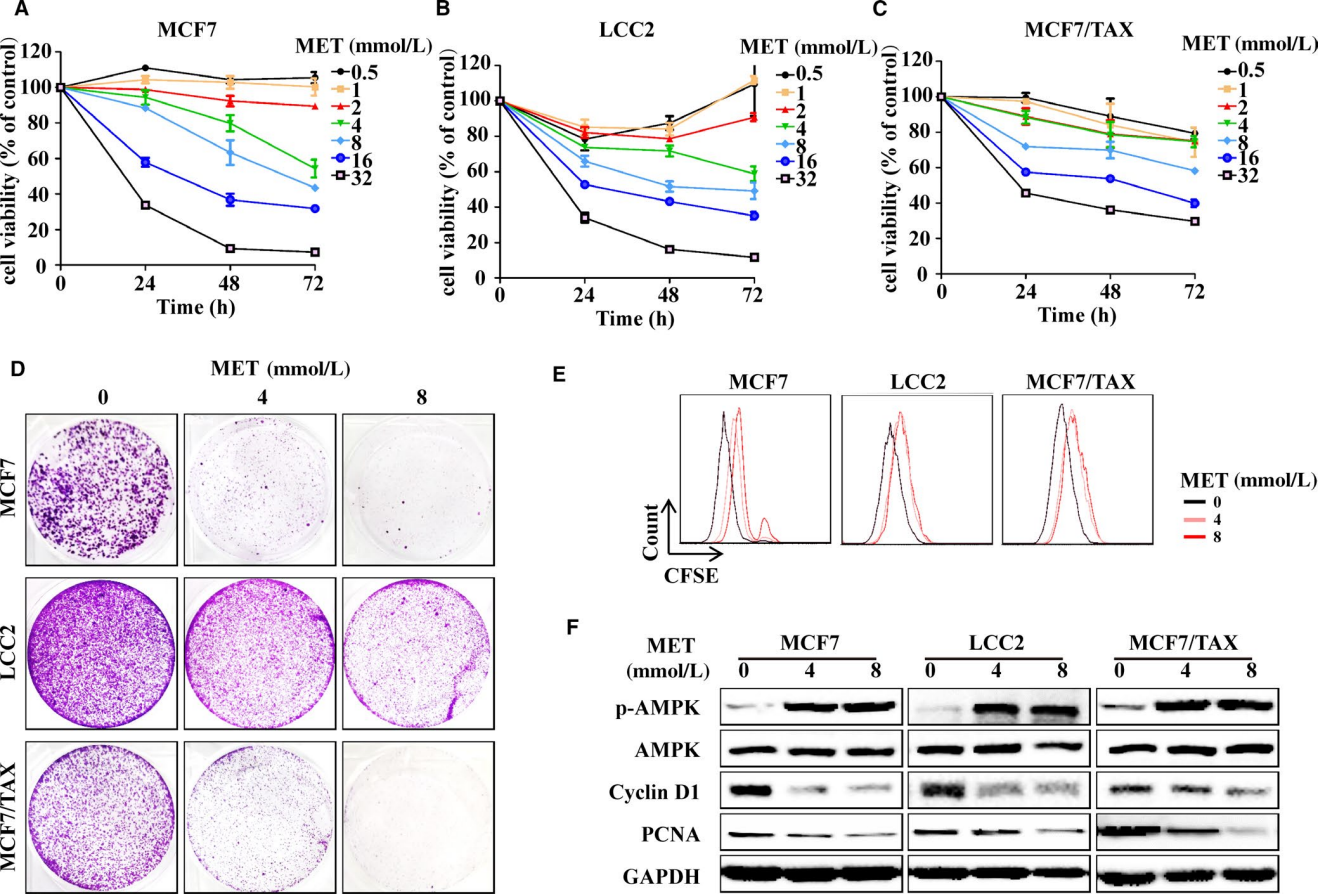
Metformin decreases survival and proliferation of drug‐sensitive and drug‐resistant breast cancer cells in vitro. A to C, MCF7, LCC2 and MCF/TAX cells were treated with MET (0, 0.5, 1, 2, 4, 8, 16 or 32 mmol/L) for 72 h, followed by measurement of cell viability using the MTT assay. D, MCF7, LCC2 and MCF/TAX cells were treated with 0, 4 or 8 mmol/L MET for 24 h, reseeded into new dishes for 14 days and then stained with crystal violet. E, Cells were stained with CFSE, treated with 0, 4 or 8 mmol/L MET and then analysed by flow cytometry. F, Cells were treated with 0, 4 or 8 mmol/L MET for 24 h and then immunoblotted for p‐AMPK, AMPK, cyclin D1 and PCNA

Recent reports showed that MET can inhibit the growth of many types of tumours, reduce the risk of cancer and improve the prognosis of patients with cancer.^15^, ^18^, ^19^ Thus, we treated drug‐sensitive breast cancer cells (MCF7), TAM‐resistant cells (LCC2) and TAX‐resistant cells (MCF/TAX) with different concentrations of MET. MET decreased cell viability in a dose‐dependent manner in all three cell types (Figure 1A‐C). However, treatment with MET alone had a similar effect as treatment with MET + TAM and MET + TAX in all three cell types (Figure S1).

Next, we used clone formation and the CFSE assay to examine the effect of MET on the proliferation of MCF7, LCC2 and MCF7/TAX cells. MET decreased the growth of all 3 cell types in a concentration‐dependent manner (Figure 1D,E). MET has well‐established effects on the phosphorylation and activation of AMPK. In agreement, our results indicated that MET increased the level of p‐APMK in drug‐resistant and drug‐sensitive breast cancer cells, and also reduced the levels of other proteins associated with cell proliferation (cyclin D1 and PCNA; Figure 1F).

**FIGURE 2:**
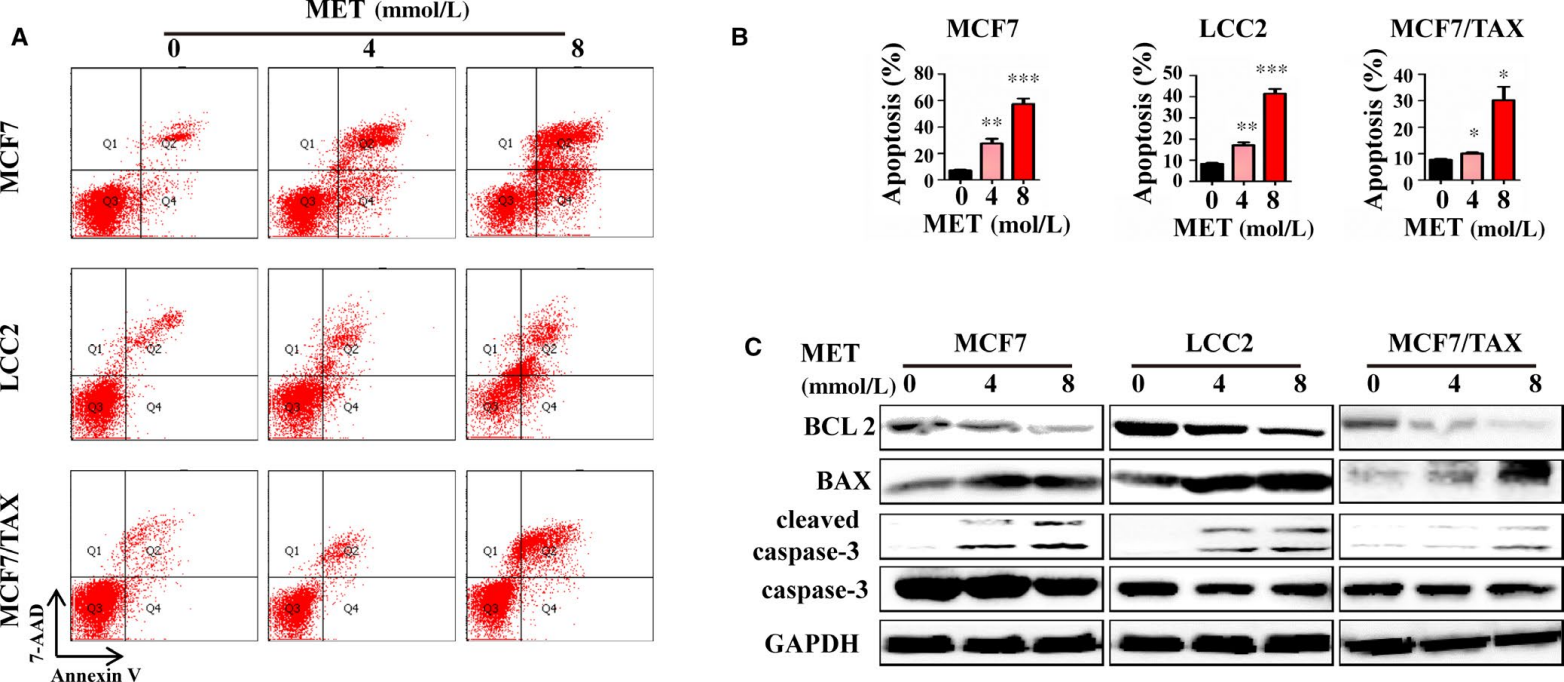
Metformin promotes apoptosis in vitro. A and B, Cells were treated with 0, 4, or 8 mmol/L MET, and apoptosis ratios were determined by FCM. C, Cells were treated with 0, 4 or 8 mmol/L MET for 24 h and then immunoblotted for apoptosis‐associated proteins (BAX, BCL2, cleaved caspase‐3 and caspase‐3)

MET treatment also increased the proportion of apoptotic drug‐sensitive and drug‐resistant breast cancer cells, induced cleavage of caspase‐3, increased BAX expression and decreased BCL2 expression (Figure 2). Thus, MET inhibited the proliferation and induced the apoptosis of drug‐sensitive and drug‐resistant breast cancer cells.

### 3.2 MET inhibits growth and metastasis of TAM‐ and TAX‐resistant breast tumours

**FIGURE 3:**
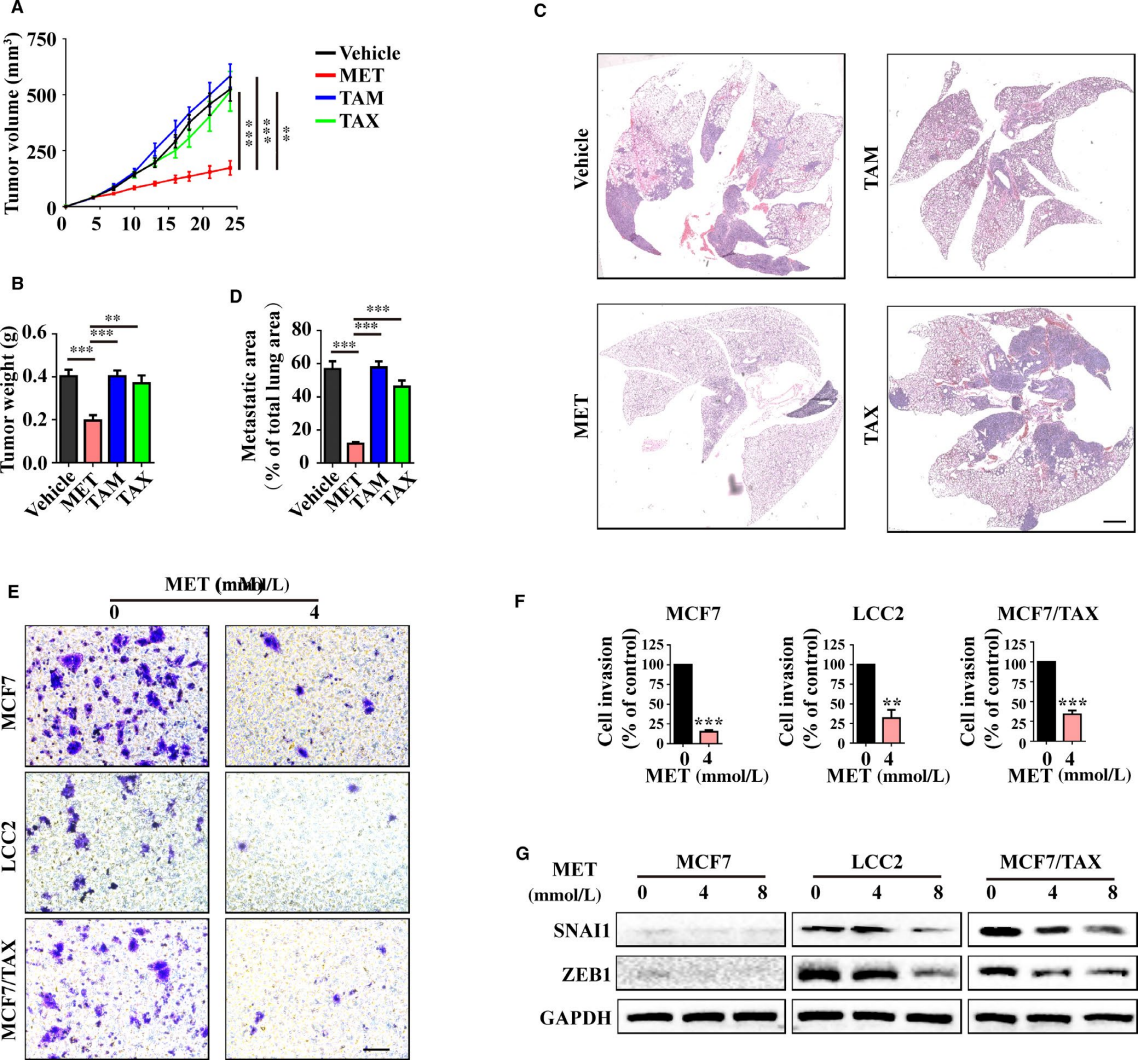
Metformin inhibits growth and invasion of breast tumours in vivo. The BALB/c mice were injected with 4T1 cells (mammary fat pad xenograft assay) and then given vehicle, MET (200 mg/kg), TAM (5 mg/kg) by gavage or TAX (20 mg/kg) by intraperitoneal injection. A and B, Tumour volume was measured every 3 days, and mice were killed on day 24 for determination of tumour weight. C and D, Metastatic area of lung tissue was determined using H&E staining (bar = 1000 μm). E and F, MCF7, LCC2 and MCF/TAX cells were given 4 mmol/L MET, and cell invasion was then determined using the transwell assay. G, Cells were treated with 0, 4 or 8 mmol/L MET for 24 h and then immunoblotted for EMT‐related transcription factors, SNAI1 and ZEB1

To further investigate the MET‐induced inhibition of drug‐resistant breast cancer cells, we injected 4T1 (murine mammary carcinoma) cells into the mammary fat pads of mice to establish an orthotopic mouse model of breast cancer. TAM and TAX treatment individually had no effect on tumour growth (Figure S2). However, MET treatment led to a significant decrease of tumour weight and volume (Figure 3A,B). These results suggest that MET can inhibit the survival of drug‐resistant breast cancer cells by itself and that combining MET with traditional anti‐cancer drugs provided no additional benefit.

Treatment of mice with MET also inhibited the lung metastasis of 4T1 tumour cells. In particular, the metastatic area was much smaller in mice that received MET (Figure 3C,D). In agreement, our in vitro studies (using the transwell assay) also indicated that MET treatment inhibited cell invasion (Figure 3E,F). Metastasis is often closely associated with the epithelial‐mesenchymal transition (EMT),^20^, ^21^ a process in which multiple signalling factors induce the expression of specific transcription factors, such as SNAI1 and ZEB1.^22^ We found that MET treatment also reduced the expression of these EMT markers in a concentration‐dependent manner (Figure 3G). These results suggest that MET can also inhibit the metastasis of tumours that are resistant to TAM and TAX.

### 3.3 MET induces YAP translocation from the nucleus to the cytoplasm

**FIGURE 4:**
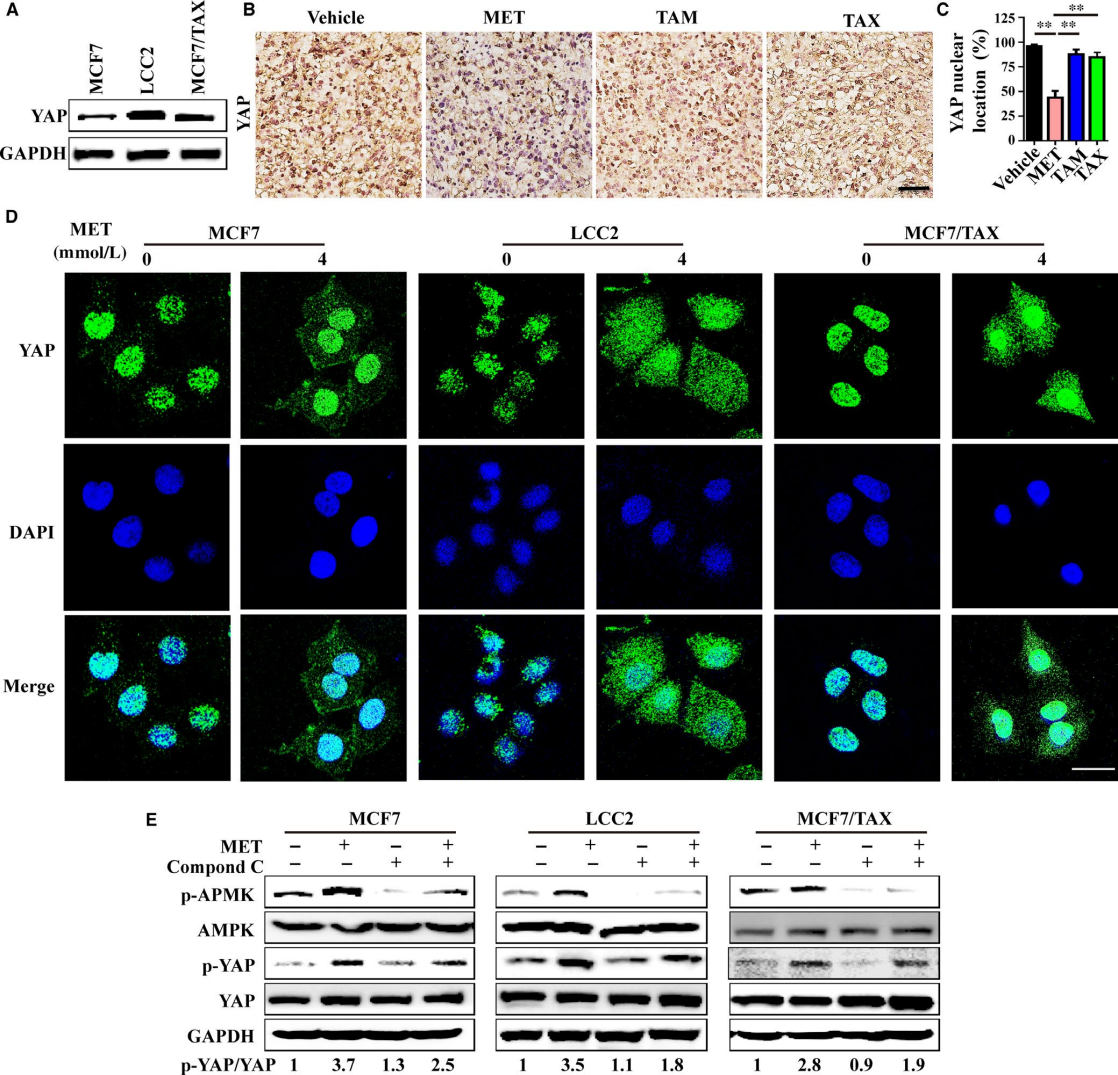
Metformin inhibition of YAP nuclear localization is independent of AMPK. A, Untreated MCF7, LCC2, and MCF/TAX cells were immunoblotted for total YAP. B and C, Mice with 4T1 tumours were given different treatments (vehicle, MET, 200 mg/kg; TAM, 5 mg/kg; TAX, 20 mg/kg) and then subjected to immunohistochemical staining for YAP, with determination of YAP nuclear localization (bar = 50 μm). D, Cells were treated with 0 or 4 mmol/L MET and then subjected to immunofluorescence staining for YAP and DAPI to determine nuclear localization (bar = 25 μm). E, Cells were treated with MET (4 mmol/L for 24 h) and/or compound C (5 μmol/L) and then immunoblotted for p‐AMPK, AMPK, p‐YAP and YAP

YAP is the downstream effector of the Hippo pathway, functions as a transcriptional co‐activator upon translocation to the nucleus, where it promotes cell proliferation and migration, and has anti‐apoptotic and other effects.^23^ Recent research suggested that YAP activation promoted drug resistance during cancer therapy.^24^, ^25^, ^26^ In agreement, we found greater total YAP expression in TAM‐ and TAX‐resistant breast cancer cells (LCC2 and MCF7/TAX) than in cells sensitive to these drugs (MCF7) (Figure 4A). Our mouse experiments also indicated that the level of nuclear YAP was down‐regulated following treatment with MET, rather than vehicle alone, TAM alone and TAX alone (Figure 4B,C).

We also examined the potential mechanisms of this response by determining YAP localization. The results indicated that MET inhibited the translocation of YAP from the cytoplasm into the nucleus, especially in LCC2 and MCF7/TAX cells (Figure 4D). In addition, MET induced the phosphorylation of YAP, and this was partly inhibited by Compound C, an APMK inhibitor (Figure 4E). These results suggest that the MET‐induced YAP inactivation occurred *via* AMPK‐dependent and APMK‐independent pathways.

### 3.4 MET activates MST and LATS kinase cascades by increasing expression and interaction with SCRIB

**FIGURE 5:**
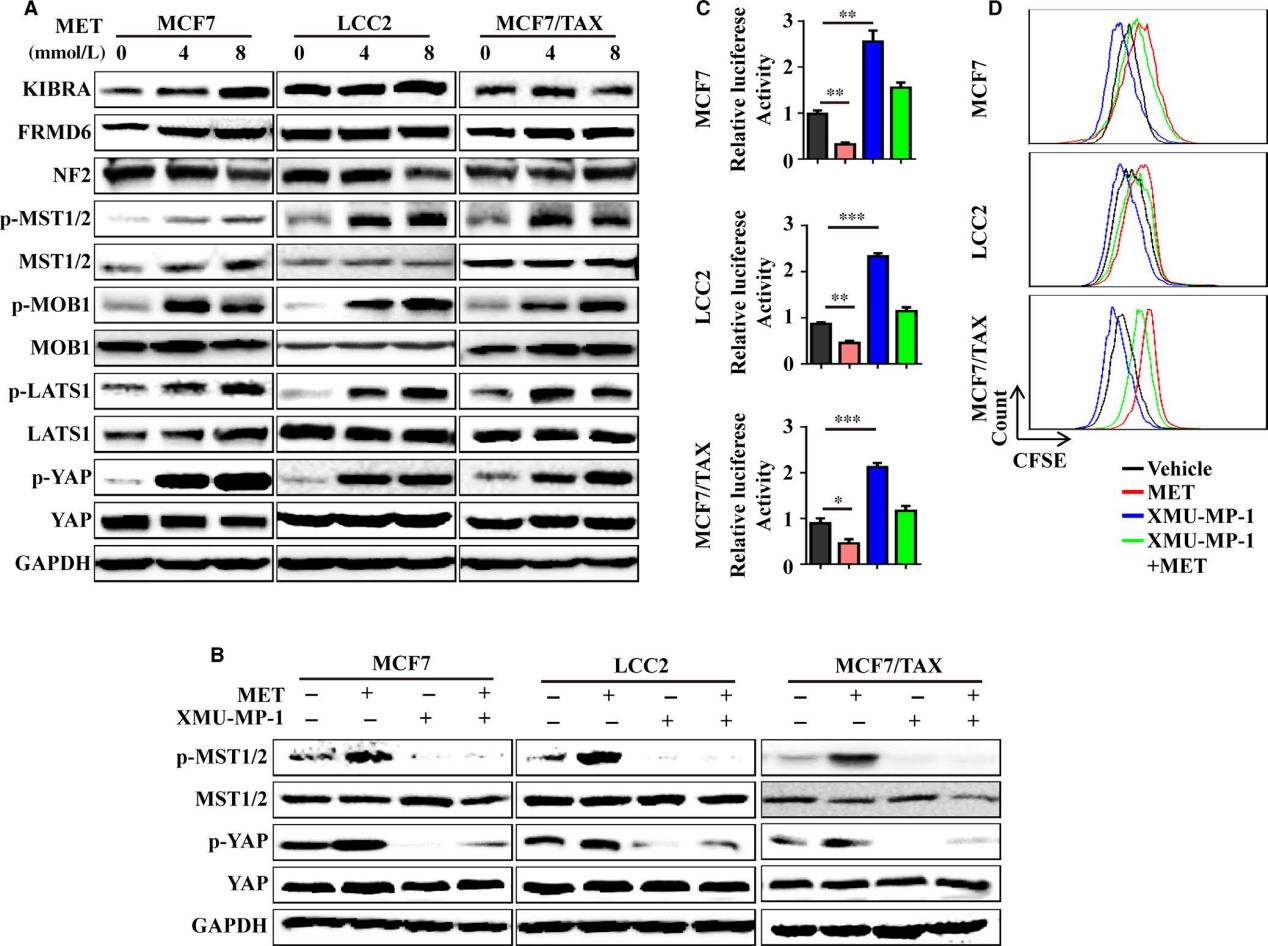
Metformin activates the Hippo pathway in drug‐resistant cells. A, MCF7, LCC2 and MCF7/TAX cells were treated with 0, 4 or 8 mmol/L MET and then immunoblotted for proteins in the Hippo pathway. B, Expression of p‐MST1/2, MST1/2, p‐YAP and YAP after treatment with MET and/or XMU/MP‐1. C, TEAD transcriptional activity was determined using a luciferase assay. D, Cell proliferation was determined after MET and/or XMU‐MP‐1 treatment of MCF7, LCC2 and MCF7/TAX cells

We measured the effect of MET treatment on the levels of major phosphorylated proteins in the Hippo pathway (p‐MST1/2, p‐MOB1 and p‐LATS1) in the same drug‐sensitive and drug‐resistant cells (Figure 5A). Previous studies reported that the MET‐induced YAP inhibition was due to MST1/2‐dependent and MST1/2‐independent effects. In particular, AMPK activation can directly inhibit YAP activation or can stabilize AMOTL1 expression without the need for MST1/2 kinases.^27^, ^28^, ^29^ Our results indicated that MET increased the level of p‐YAP and TEAD transcriptional activity and reduced cell proliferation and that XMU‐MP‐1 (an inhibitor of MST1/2 kinase) blocked these effects (Figure 5B‐D). This suggests that the MET‐induced YAP phosphorylation depended on MST1/2. However, there was increased expression of the classical Hippo pathway upstream proteins (KIBRA and FRMD6) in MCF7 cells, but not in LCC2 and MCF/TAX cells (Figure 5A). Thus, it is possible that other MST1/2‐dependent upstream regulators participated in the MET‐induced activation of the Hippo pathway in these drug‐resistant cells.

**FIGURE 6:**
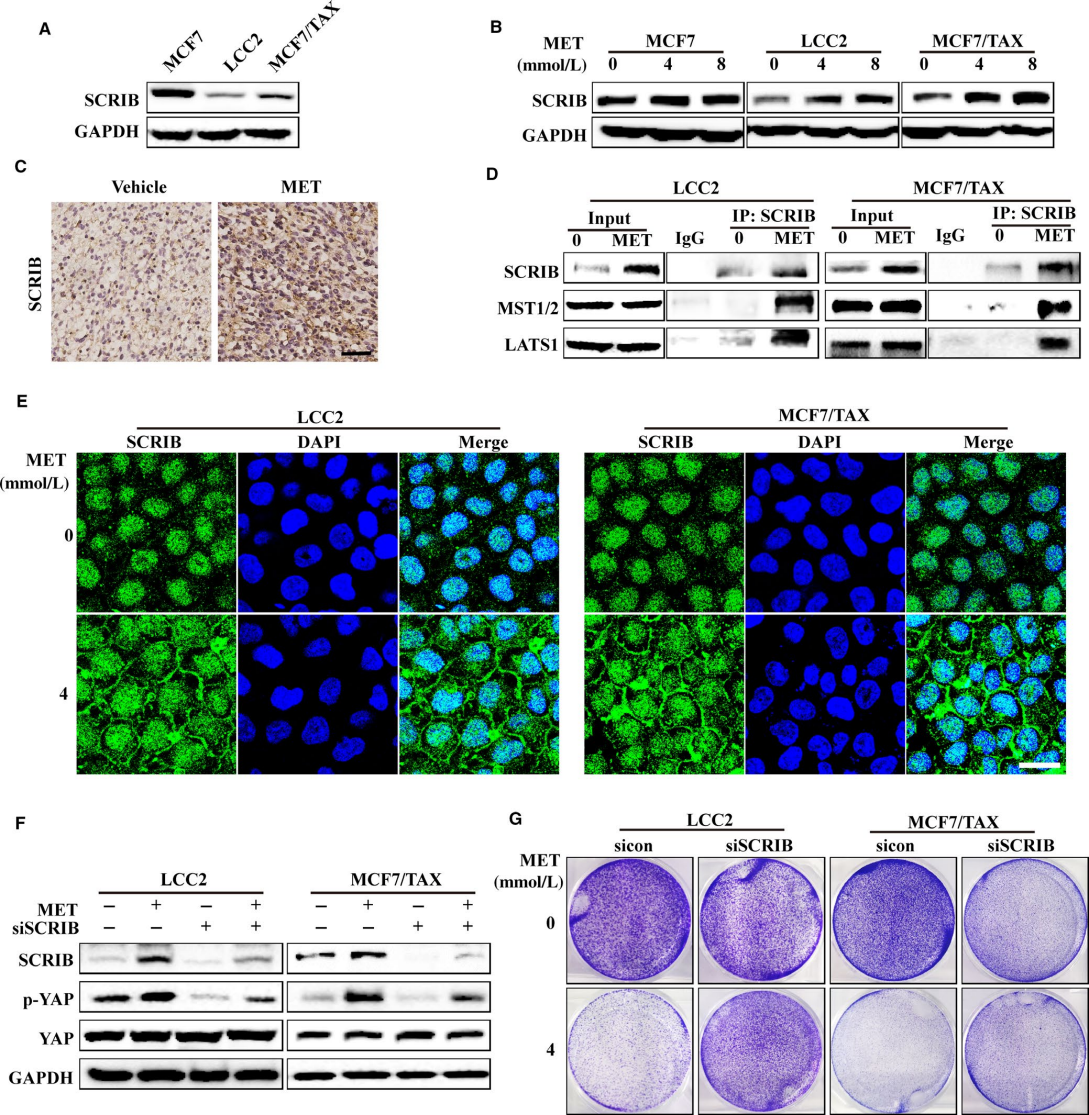
Metformin activates the Hippo pathway by increasing the expression and membrane localization of SCRIB in vitro. A, SCRIB expression in untreated cells. B, Cells were treated with 0, 4 or 8 mmol/L MET and then subjected to immunoblotting for SCRIB. C, Mice with 4T1 tumours were given different treatments (vehicle or MET, 200 mg/kg) and then subjected to immunohistochemical staining for SCRIB (bar = 50 μm) D, Co‐immunoprecipitation of SCRIB with MST and LATS after MET treatment of drug‐resistant cells. E, Drug‐resistant cells were treated with 0 or 4 mmol/L MET and then subjected to immunofluorescence staining for SCRIB and DAPI to determine nuclear localization (bar = 25 μm). Western blot analysis (F) and colony formation assay (G) of p‐YAP expression after siRNA‐mediated SCRIB knockdown and treatment with 0 or 4 mmol/L MET

Besides the classical upstream regulators, recent research has identified many new regulators of the Hippo pathway, such as apical‐basal polarity proteins (eg LKB1, SCRIB, CRB3, DLG5 and PTPN14), planar cell polarity proteins (eg FAT‐4, DCHS1/2 and ZYX) and other proteins (eg TAOK1‐3, RASSF1‐6, β‐TRCP and 14‐3‐3).^30^, ^31^, ^32^, ^33^ Our examination of untreated cells indicated significantly lower expression of the cell polarity protein SCRIB in LCC2 and MCF/TAX cells than in MCF7 cells (Figure 6A). Interestingly, MET treatment increased the expression of SCRIB in the two drug‐resistant cells (LCC2 and MCF/TAX) and in mouse tumours, but only had a weak effect in drug‐sensitive cells (MCF7; Figure 6B,C). MET treatment tended to increase the mRNA level of *SCRIB*, but this increase was not statistically significant (Figure S3). A co‐immunoprecipitation assay showed that MET treatment led to increased interaction of SCRIB with MST1/2 and LATS1 in the drug‐resistant cell lines (Figure 6D). In addition, MET treatment led to increased membrane localization of scribble in LCC2 cells and MCF/TAX cells (Figure 6E). MET‐induced YAP phosphorylation and inhibition of cell proliferation were abrogated after knockdown of SCRIB (Figure 6F,G).

**FIGURE 7:**
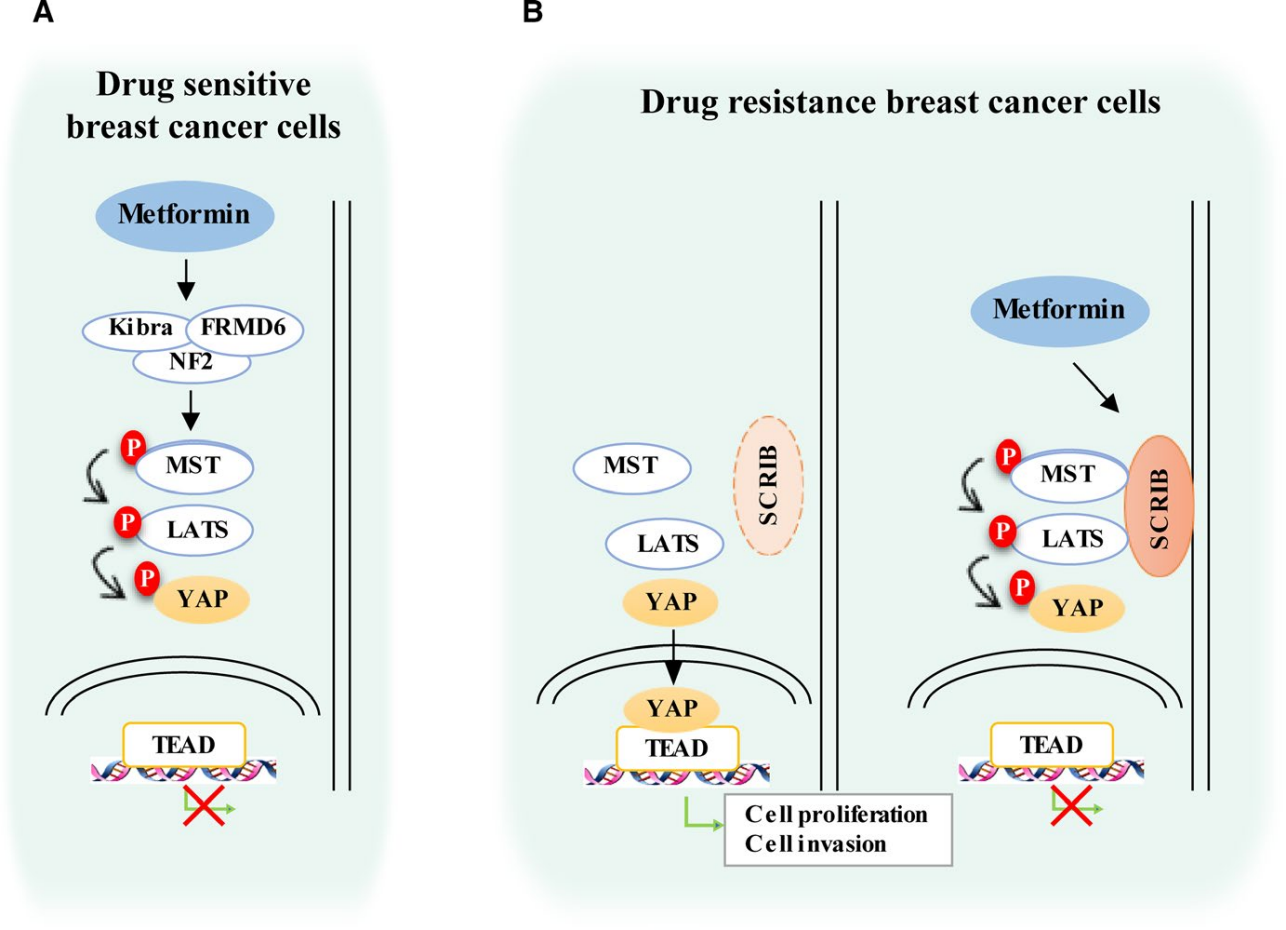
Proposed model of metformin‐induced activation of the Hippo pathway in drug‐sensitive and drug‐resistant breast cancer cells. A, In drug‐sensitive cancer cells (MCF7), MET activates the Hippo pathway by increasing the expression KIBRA and FRMD6, which increases the phosphorylation of YAP and prevents its translocation and binding to TEAD in the nucleus. B, In drug‐resistant cancer cells (LCC2 and MCF7/TAX), MET activates the Hippo pathway by increasing the expression and plasma membrane localization of SCRIB, which increases the phosphorylation of YAP and prevents its translocation and binding to TEAD in the nucleus

Our analysis of drug‐sensitive breast cancer cells indicated that MET inhibited cell proliferation and invasion by increasing the expression of the classical Hippo upstream regulators, KIBRA and FRMD6 (Figure 7A). Our analysis of drug‐resistant breast cancer cells indicated that MET increased SCRIB expression, which then recruited MST1/2 and LATS1 to the plasma membrane, leading to YAP phosphorylation and its retention within the cytoplasm, and finally to inhibition of cell proliferation and invasion (Figure 7B). Thus, the MET‐induced activation of the Hippo pathway occurs because of the increased expression and plasma membrane localization of SCRIB, which inhibits YAP translocation to the nucleus.

## 4 DISCUSSION

Drug resistance is a major adverse effect that can occur during breast cancer therapy.^3^, ^5^ In this study, we propose a new treatment that can overcome drug resistance, in which MET activates the Hippo pathway of drug‐resistant cells by increasing the expression of SCRIB (Figure 7). In particular, we found that MET up‐regulated SCRIB, leading to YAP phosphorylation and localization to the cytoplasm, and ultimately to inhibition of the growth and invasion of TAM‐ and TAX‐resistant breast cancer cells.

YAP is a Hippo pathway effector that stimulates drug resistance to many types drugs used in cancer therapy.^8^, ^34^ In our study, we also noticed higher YAP expression in TAM‐ and TAX‐resistant breast cancer cells (LCC2 and MCF7/TAX) than in drug‐sensitive breast cancer cells (MCF7). Inhibition of the expression and activation of YAP is a major approach used to overcome YAP‐dependent drug resistance.^35^, ^36^ Previous research showed that silencing of YAP expression enhanced the in vitro sensitivity to MEK and RAF inhibitors in lung cancer, colon cancer, melanoma, pancreatic cancer and thyroid cancer.^26^ Activation of YAP also plays a crucial role in the drug resistance of oesophageal cancer and in the BRAF inhibitor resistance of melanoma cells.^25^ YAP‐induced up‐regulation of the epidermal growth factor receptor (EGFR) plays an important role in conferring drug resistance to oesophageal cancer cells. Thus, targeting the YAP‐EGFR axis may be more efficacious than targeting EGFR alone as a treatment of oesophageal cancer.^37^ Here, we found MET treatment decreased the activation of YAP by increasing the level of p‐YAP in drug‐resistant breast cancer cells.

Recent research reported that MET (an AMPK inducer) increased the phosphorylation of YAP by promoting APMK kinase activity or by stabilizing AMOTL1 expression, effects that do not involve the classical Hippo upstream kinase MST1/2.^27^, ^28^ Here, we found that MET reduced the survival of breast cancer cells by an AMPK‐independent pathway. We also identified different effectors of MET‐induced activation of the Hippo pathway in drug‐sensitive cells (MCF7) and drug‐resistant cells (LCC2 and MCF7/TAX). MET treatment of MCF7 cells led to increased expression of the well‐known upstream regulators, KIBRA and FRDM6; however, MET treatment of LCC2 and MCF7/TAX cells led to no significant changes of the classical Hippo regulators.

Our previous studies indicated that cell polarity proteins can regulate the Hippo pathway and that these proteins (eg CRB3 and DLG5) are down‐regulated in drug‐resistant cells.^38^, ^39^ This led us to focus on other regulators of the Hippo pathway in our examination of drug‐resistant cancer cells. We observed greater downregulation of the cell polarity protein SCRIB in drug‐resistant cells than in drug‐sensitive cells. SCRIB is a core member of the basolateral polarity complex, which functions in establishment of epithelial cell polarity, and it is mis‐localized or down‐regulated in many cancers.^21^, ^40^ The basolateral localization of SCRIB led to interactions with core kinases of the Hippo pathway (MST and LATS), and this contributes to activation of the Hippo pathway.^41^, ^42^ In contrast to drug‐sensitive cells, MET treatment led to notable up‐regulation of SCRIB in drug‐resistant cells. More specifically, we found that MET increased the expression and membrane localization of SCRIB in drug‐resistant cells, preserving the interaction of SCRIB with MST and LATS. This led to YAP phosphorylation and inhibition of its translocation into the nucleus, consequently reducing the survival and invasiveness of drug‐resistant breast cancer cells. Our future work will examine other regulators in the MET‐induced activation of the Hippo pathway.

The development of new drugs can be expensive and time‐consuming, and it is necessary to examine side effects and patient tolerability. The development of toxicities not predicted by preclinical work can greatly prolong the development time.^43^ Thus, repurposing a well‐established drug, such as MET, obviates these limitations. MET has been used clinically for many years and has acceptable safety and tolerability. MET is the first‐line therapy for all patients who are newly diagnosed with type 2 diabetes^12^ and is safe and tolerable. However, the safety and tolerability of MET at doses needed to treat cancer patients requires confirmation. Our results, in combination with other recent research, suggest that MET should be considered for treatment of drug‐resistant breast cancer.

## CONFLICT OF INTEREST

The authors declare no conflict of interest.

## AUTHOR CONTRIBUTIONS

Jie Liu and Peijun Liu participated in the design of the study and drafting the manuscript. Jie Liu and Juan Li carried out most of the experiments and analysed the data. He Chen and Ruiqi Wang prepared cancer tissues and sections for staining. Pingping Li and Yi Miao participated in the statistical analysis. All authors have read and approved the final manuscript.

## Supporting information

Figure S1

Figure S2

Figure S3

Supplementary Material

## Data Availability

The data that support the findings of this study are available from the corresponding author upon reasonable request.
