# Supplementary figures and images for: Metformin suppresses proliferation and invasion of drug‐resistant breast cancer cells by activation of the Hippo pathway
Source: J Cell Mol Med. 2020 Apr 12;24(10):5786–96. doi: 10.1111/jcmm.15241 (PMC7214175; doi:10.1111/jcmm.15241)

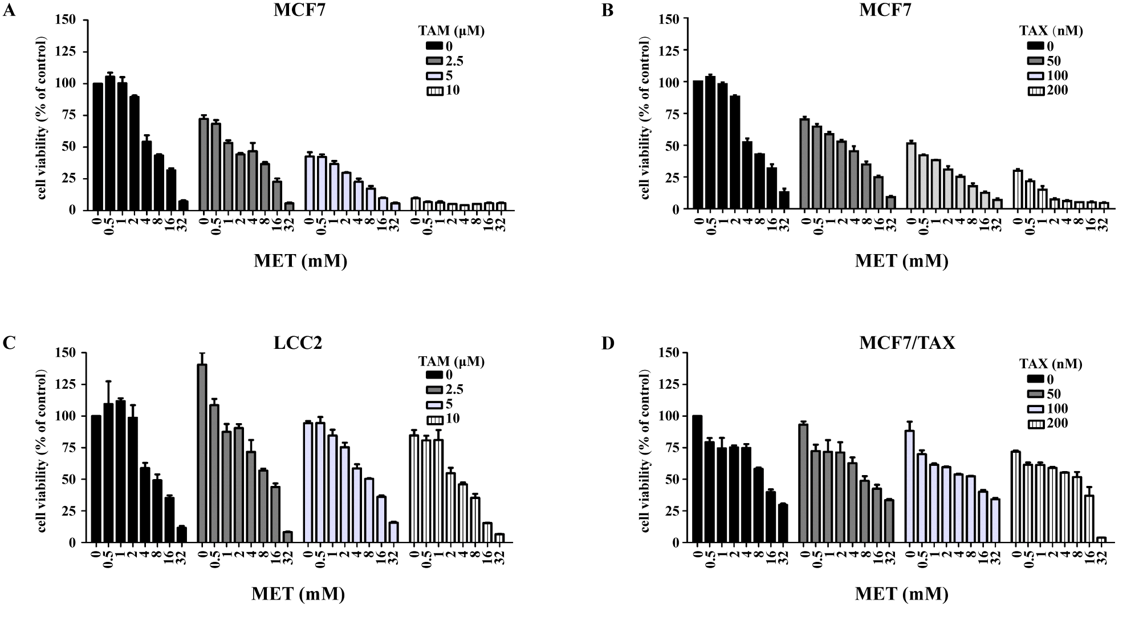

Supplement: Supplementary file 1 — Figure S1 [file JCMM-24-5786-s001.tif]

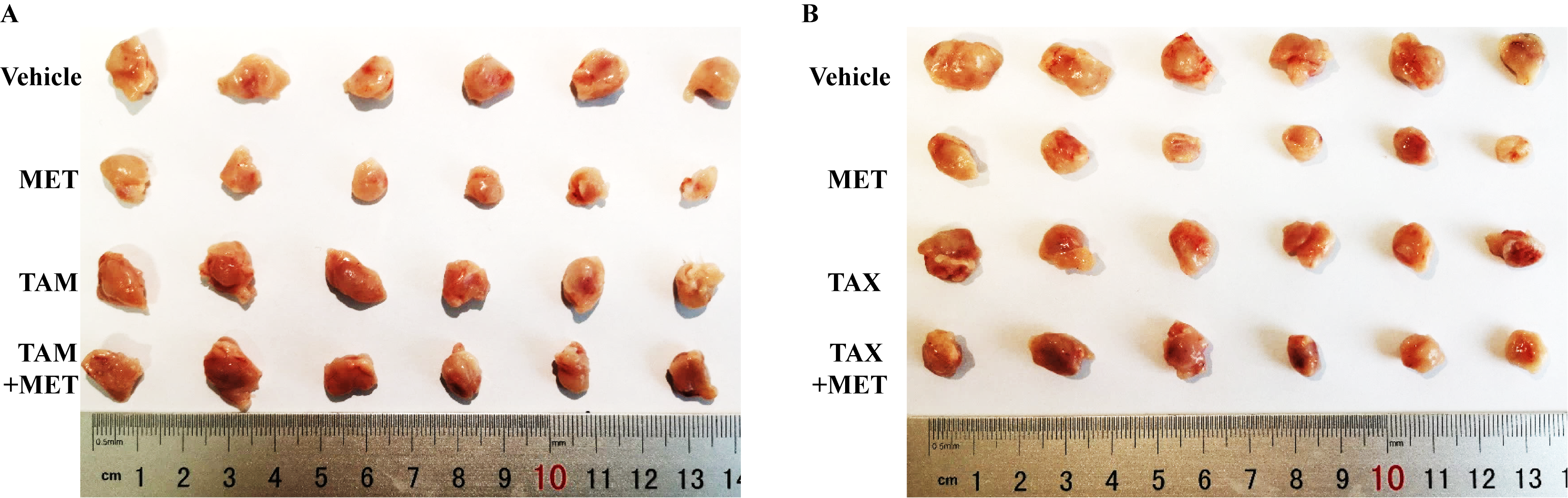

Supplement: Supplementary file 2 — Figure S2 [file JCMM-24-5786-s002.tif]

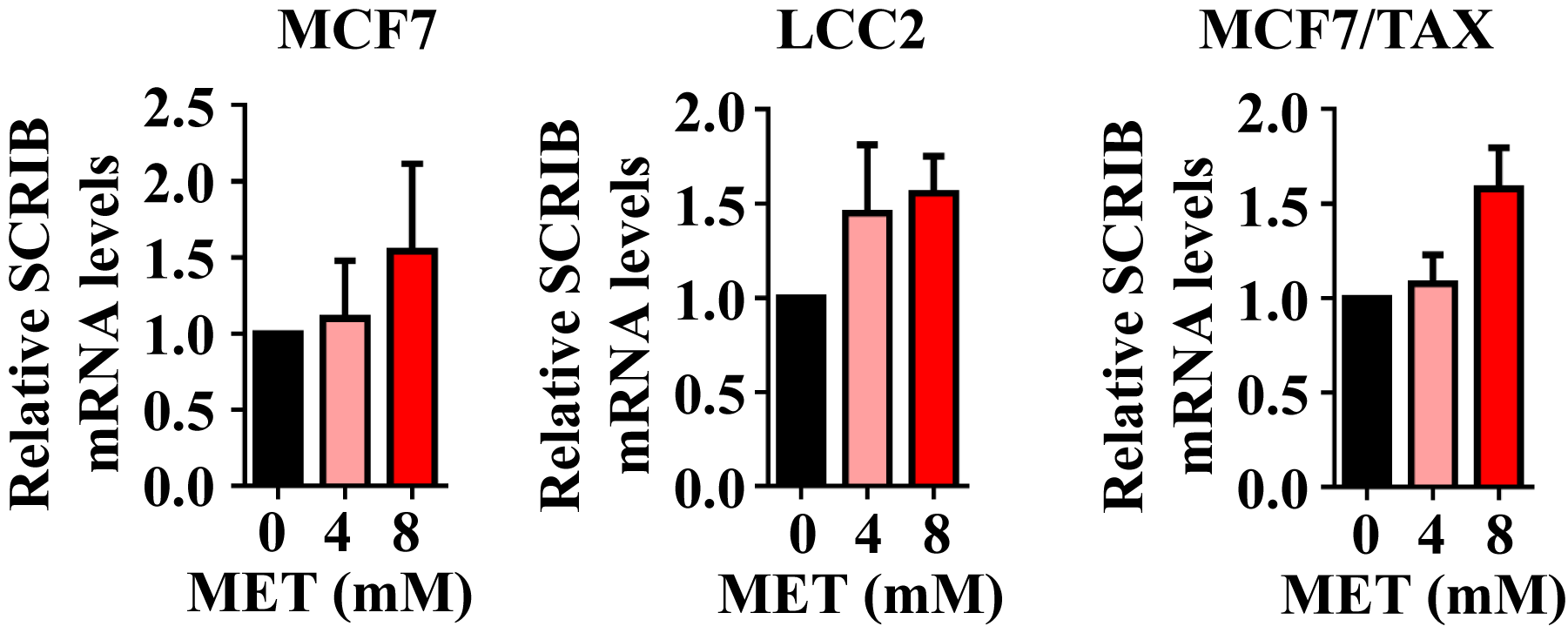

Supplement: Supplementary file 3 — Figure S3 [file JCMM-24-5786-s003.tif]
