# Supplementary material for: Metformin suppresses proliferation and invasion of drug‐resistant breast cancer cells by activation of the Hippo pathway
Source: J Cell Mol Med. 2020 Apr 12;24(10):5786–96. doi: 10.1111/jcmm.15241 (PMC7214175; doi:10.1111/jcmm.15241)
Supplement: Supplementary file 4 — Supplementary Material [file JCMM-24-5786-s004.docx]

**Supporting Information:**

**Figure s1. Addition of tamoxifen or paclitaxel does not affect survival of breast cancer cells treated with metformin *in vitro*.**

**A and C**, MCF7 and LCC2 cells were treated with MET (0, 0.5, 1, 2, 4, 8, 16, or 32 mM), TAM (0, 2.5, 5, or 10 μM), or MET+TAM for 72 h, followed by measurement of cell viability using the MTT assay. **B and D**, MCF7 and MCF/TAX cells were treated with MET (0, 0.5, 1, 2, 4, 8, 16, or 32 mM), TAX (0, 50, 100, or 200 nM), or MET+TAX for 72 h, followed by measurement of cell viability.

**Figure s2. Metformin inhibits growth of breast tumors *in vivo*.**

**A**, Breasts of BALB/c mice were injected with 4T1 cells (mammary fat pad xenograft assay) and then given vehicle, MET (200 mg/kg), TAM (5 mg/kg), or MET+TAM. **B**, The same experiment was performed with mice given vehicle, MET (200 mg/kg), TAX (20 mg/kg), or MET+TAX.

**Figure s3. MET treatment tended to increase the mRNA level of *SCRIB*, but this increase was not statistically significant**

Cells were treated with 0, 4, or 8 mM MET, and SCRIB mRNA level was then using qRT‐PCR.
